# Impact of relative dose intensity on bone marrow suppression induced by S-1: retrospective observational study

**DOI:** 10.1186/s40780-018-0127-x

**Published:** 2018-12-03

**Authors:** Toshinori Hirai, Ryuichi Ogawa, Ryosuke Yamaga, Toshimasa Itoh

**Affiliations:** 1 Department of Pharmacy, Tokyo Women’s Medical University Medical Center East, 2-1-10, Nishiogu, Arakawa-ku, Tokyo, 116-0011 Japan; 2 Department of Pharmacotherapy, Meiji Pharmaceutical University, Tokyo, Japan

**Keywords:** S-1, Bone marrow suppression, Relative dose intensity, Lymphocytopenia

## Abstract

**Background:**

S-1 (a combination of tegafur, gimeracil, and oteracil) is used to treat various cancers. Bone marrow suppression is a dose-limiting toxicity of S-1. The relationship between relative dose intensity (RDI) and bone marrow suppression has not been investigated. Hence, we aimed to elucidate the threshold for RDI to identify bone marrow suppression induced by S-1.

**Methods:**

In this retrospective cohort study, patients who initiated S-1 treatment at Tokyo Women’s Medical University, Medical Center East between June 2015 and June 2017 were included. Bone marrow suppression induced by S-1 was assessed using Common Terminology Criteria for Adverse Events version 4.0. The relationships between grade 3 or higher bone marrow suppression induced by S-1 and RDIs (i.e., 70, 75, and 80%) were investigated using the multivariate Cox proportional hazard model.

**Results:**

We identified 143 patients in this study. The median RDI was 78.8%. Bone marrow suppression induced by S-1 developed in 19 (13.3%) patients. The multivariate Cox proportional hazard model revealed that grade ≥ 2 lymphocytopenia was associated with bone marrow suppression induced by S-1 regardless of the threshold for RDI. In addition, RDI > 75% [hazard ratio (HR) = 1.71, *p* < 0.05] and RDI > 80% (HR = 1.65, *p* < 0.05) were associated with bone marrow suppression induced by S-1.

**Conclusions:**

Reduced dose of S-1 still has the risk of developing bone marrow suppression. Clinicians should assess RDI to identify high risk patients with bone marrow suppression induced by S-1.

## Introduction

S-1 is used for the treatment of gastric, colorectal, and lung cancer [1–3]. S-1 consists of tegafur, gimeracil, and oteracil in a molar ratio of 1:0.4:1 [4]. Tegafur is a prodrug of 5-fluorouracil (5-FU), whereas gimeracil and oteracil act to increase the concentration of 5-FU and reduce gastrointestinal toxicity by inhibiting dihydropyridine dehydrogenase and orotate phosphoribosyltransferase, respectively [5, 6]. Because the pharmacokinetics of 5-FU and gimeracil depends on creatinine clearance, 5-FU can accumulate in patients with renal impairment [7]. Thus, S-1 dosages are adjusted according to creatinine clearance and body surface area (BSA) [8].

Bone marrow suppression is a dose-limiting toxicity of S-1 that can result in dose reduction, prolongation of the washout period, and discontinuation of S-1. In fact, the occurrence of bone marrow suppression depends on the level of exposure to chemotherapeutic agents [9]. Relative dose intensity (RDI) is a marker of the exposure of chemotherapeutic agents, and RDI > 80% is associated with anti-tumor effects of S-1 [10, 11]. However, there are limited data regarding the relationship between bone marrow suppression induced by S-1 and RDI. Therefore, we hypothesize that the relevance between the incidence of bone marrow suppression and RDI will be helpful to distinguish clinically high-risk patients with bone marrow suppression induced by S-1.

Hence, the aim of this study was to investigate the relationship between bone marrow suppression induced by S-1 and threshold for RDI.

## Patients and methods

### Study design and patients

This study was a single-center retrospective cohort study. All patients who initiated S-1 treatment between June 2015 and June 2017 at Tokyo Women’s Medical University, Medical Center East, were included. Patients who lacked data on complete blood count or RDI due to unknown BSA were excluded. We could not calculate the sample size before the study began because the study was retrospective and unable to retrieve the intended number of patients from sufficient study population who initiated S-1 treatment in our hospital. The study protocol was approved by the institutional review board at Tokyo Women’s Medical University Hospital (#4467) prior to initiation of the study.

### Data collection

We extracted patients’ demographic data from electronic medical records. Their demographic data included background information (gender, age, height, body weight, body mass index [BMI], and BSA), clinical laboratory data (white blood cell [WBC] count, absolute neutrophil count [ANC], absolute lymphocyte count [ALC], hemoglobin [Hb], platelet count [Plt], aspartate transaminase [AST], alanine aminotransferase [ALT], total bilirubin, serum albumin, serum creatinine, and estimated glomerular filtration rate [eGFR]), indication for S-1 treatment (gastrointestinal, lung, breast, or the other cancers), clinical cancer stage (≥III), details of S-1 treatment (RDI and the presence of combination chemotherapy), and prior history of cytotoxic chemotherapy. eGFR was calculated by prediction equation optimized for Japanese population [12]. RDI was calculated as the ratio of actual dose intensity to planned dose intensity.

### Outcome

Bone marrow suppression induced by S-1 was defined as the occurrence of grade 3 or higher hematologic adverse events during S-1 treatment in accordance with the Common Terminology Criteria for Adverse Events (CTCAE) version 4.0 [13]; leukopenia (< 20.0 × 10^2^/μL), neutropenia (< 10.0 × 10^2^/μL), lymphocytopenia (< 5.0 × 10^2^/μL), anemia (< 8.0 g/dL), and thrombocytopenia (< 5.0 × 10^4^/μL). The follow-up duration was defined as the time from initiation of S-1 treatment to termination of S-1 treatment or the occurrence of bone marrow suppression.

### Data analysis

Kaplan–Meier curves for the cumulative incidence of bone marrow suppression induced by S-1 were stratified by RDI and compared using the log-rank test. The threshold for RDI was examined using three definitions (> 70% or ≤ 70, > 75% or ≤ 75, and > 80% or ≤ 80%).

A multivariate Cox proportional hazard model was used to assess the relationship between bone marrow suppression induced by S-1 and RDI. The dependent and independent variables were defined as grade ≥ 3 of bone marrow suppression induced by S-1 and patient characteristics at the initiation of S-1 treatment, respectively. Grade ≥ 2 of bone marrow suppression (i.e., leukopenia [< 30.0 × 10^2^ /μL], neutropenia [< 15.0 × 10^2^ /μL], lymphocytopenia [< 8.0 × 10^2^ /μL], anemia [< 10.0 g/dL], and thrombocytopenia [< 7.5 × 10^4^ /μL] defined by CTCAE version 4.0 [13]) at the initiation of S-1 treatment were categorized as binominal variables. Indications for S-1 treatment were categorized as gastrointestinal cancer and non-gastrointestinal cancer. Three definitions were used for the thresholds for RDI (> 70% or ≤ 70, > 75% or ≤ 75, and > 80% or ≤ 80%) and three final models were constructed accordingly.

We selected potential independent variables with *p* < 0.1 by univariate Cox proportional hazard analysis for multivariate Cox proportional hazard analysis. When there was multicollinearity between any of the variables, we selected one of them in the light of clinical relevance. Independent variables for the multivariate Cox proportional hazard model were determined using a stepwise forward selection method according to the Akaike information criterion.

Continuous data are represented as mean and standard deviation (SD) or median and interquartile range (IQR), and categorical data are represented as percentage. Hazard ratios (HR) and the 95% confidence interval (95% CI) for bone marrow suppression induced by S-1 were calculated by Cox proportional hazard analysis. A *p* < 0.05 was regarded as statistically significant unless otherwise noted. Statistical analyses were performed using JMP® pro 13 (SAS Institute Inc., Cary, NC, USA).

## Results

### Study patients

In total, 200 patients have initiated S-1 treatment during study period at Tokyo Women’s Medical University, Medical Center East. We excluded 47 patients who lacked data on complete blood count and 10 patients who could not calculate RDI. Thus, we identified 143 patients who fulfilled the inclusion criteria. Their demographic data are shown in Table 1. Males accounted for 92 (64.3%) of all patients; the mean age (SD) was 67.2 (10.6) years. The numbers of patients with grade ≥ 2 leukopenia, neutropenia, lymphocytopenia, and anemia at initiation of S-1 treatment were 6 (4.2%), 5 (3.5%), 7 (4.9%), and 12 (8.4%), respectively. No patients had grade ≥ 2 thrombocytopenia at the initiation of S-1 treatment. The majority of patients had gastrointestinal cancer. The median RDI (IQR) was 78.8 (70.7–85.8) %. Patients with RDI > 70, > 75, and > 80% were 113 (79.0%), 85 (59.4%), and 68 (47.6%), respectively. There were 52 (36.4%) and 51 (35.7%) patients who had prior history of cytotoxic chemotherapy and who received combination chemotherapy, respectively, in overall study population. When we stratified the population by RDI of 70, 75, and 80%, patients categorized in the higher RDI showed significantly higher rate of combination therapy in every cutoff RDI (70%: 40.7% vs 16.7, 75%: 43.5% vs 24.1, 80%: 50.0% vs 22.7%). Besides, there were no significant interactions with the prior history of cytotoxic chemotherapy.

**Table 1:** Demographic data

Characteristics (Number = 143)	Values
Patient background	
Male (%)	92 (64.3)
Age (years)	67.2 ± 10.6
Height (cm)	160.7 ± 9.3
Body weight (kg)	53.5 ± 11.3
BMI (kg/m^2^)	20.7 ± 3.8
BSA (m^2^)	1.54 ± 0.18
Clinical laboratory data	
WBC (×10^2^/μL)	53.0 [44.0–70.0]
ANC (×10^2^/μL)	35.0 [23.0–54.0]
ALC (×10^2^/μL)	25.3 [14.8–33.0]
Hb (g/dL)	11.9 ± 1.5
Plt (×10^4^/μL)	21.4 [17.7–26.5]
AST (IU/L)	22.0 [18.0–31.0]
ALT (IU/L)	16.0 [11.0–24.0]
Total bilirubin (mg/dL)	0.6 [0.5–0.9]
Serum albumin (g/dL)	3.8 [3.5–4.1]
Serum creatinine (mg/dL)	0.7 [0.6–0.8]
eGFR^a^ (mL/min/1.73 m^2^)	78.1 ± 21.5
Indication for S-1 treatment	
Gastrointestinal cancer (%)	110 (76.9)
Lung cancer (%)	9 (6.3)
Breast cancer (%)	9 (6.3)
Other cancers (%)	15 (10.5)
Clinical cancer stage ≥III (%)	96 (67.1)
Details of S-1 treatment	
RDI^b^ (%)	78.8 [70.7–85.8]
RDI^b^ > 70% (%)	113 (79.0)
RDI^b^ > 75% (%)	85 (59.4)
RDI^b^ > 80% (%)	68 (47.6)
Presence of combination chemotherapy (%)	51 (35.7)
Prior history of cytotoxic chemotherapy (%)	52 (36.4)

Continuous data are expressed as mean ± SD or median [IQR] as appropriate. Categorical data are expressed as number (%)

*SD* standard deviation, *IQR* interquartile range, *BMI* body mass index, *BSA* body surface area, *WBC* white blood cell count, *ANC* absolute neutrophil count, *ALC* absolute lymphocyte count, *Hb* hemoglobin, *Plt* platelet count, *AST* aspartate transaminase, *ALT* alanine aminotransferase, *eGFR* estimated glomerular filtration rate, *RDI* relative dose intensity

^a^eGFR was calculated using a prediction equation

^b^RDI is the ratio of the actual dose intensity to the planned dose intensity

### Outcome

Bone marrow suppression induced by S-1 was identified in 19 (13.3%) patients. Grade ≥ 3 leucopenia developed in 2 (10.5%) of the 19 patients. Grade ≥ 3 neutropenia, lymphocytopenia, and anemia developed in 7 (36.8%) of the 19 patients. No patient had grade ≥ 3 thrombocytopenia. The median (IQR) follow-up duration was 42 (14–175) days.

**Fig. 1:**
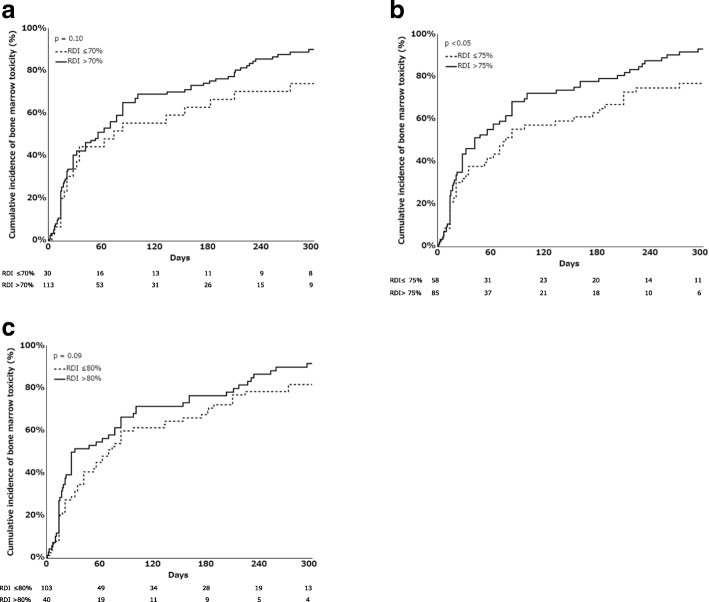
Kaplan–Meier curves for the cumulative incidence of bone marrow suppression induced by S-1. The curves were analyzed using a log-rank test. The x- and y-axes represent the number of days after initiation of S-1 and the cumulative incidence of bone marrow suppression induced by S-1, respectively. Number at risk was shown below x- axis. RDI is the ratio of the actual dose intensity to the planned dose intensity. RDI, Relative dose intensity. **a.** Kaplan–Meier curves for the cumulative incidence of bone marrow suppression induced by S-1 in patients with RDI > 70% and RDI ≤70% (Model 1). Solid and dotted lines represent RDI > 70% and RDI ≤70%, respectively. **b.** Kaplan–Meier curves for the cumulative incidence of bone marrow suppression induced by S-1 in patients with RDI > 75% and RDI ≤75% (Model 2). Solid and dotted lines represent RDI > 75% and RDI ≤75%, respectively. **c.** Kaplan–Meier curves for the cumulative incidence of bone marrow suppression induced by S-1 in patients with RDI > 80% and RDI ≤80% (Model 3). Solid and dotted lines represent RDI > 80% and RDI ≤80%, respectively

RDI > 70% did not significantly affect the cumulative incidence of bone marrow suppression induced by S-1 (Model 1, *p* = 0.10; Fig. 1a). Patients with RDI > 75% had a higher cumulative incidence of bone marrow suppression induced by S-1 compared to RDI ≤75% (Model 2, *p* < 0.05; Fig 1b). RDI > 80% had no effect on the cumulative incidence of bone marrow suppression induced by S-1 (Model 3, *p* = 0.09; Fig. 1c).

### Data analysis

Univariate Cox proportional hazard model analysis revealed that grade ≥ 2 lymphocytopenia, total bilirubin, RDI > 70%, RDI > 75%, RDI > 80%, and presence of combination chemotherapy were associated with bone marrow suppression induced by S-1 (Table 2). Using a stepwise forward selection method, three final models were determined stratified by three thresholds for RDI. Bone marrow suppression induced by S-1 was associated with grade ≥ 2 lymphocytopenia in all three final models (Table 3). Although RDI > 70% (Model 1) has no significant effect on bone marrow suppression induced by S-1, RDI > 75 and > 80% (Model 2 and 3) have a significant effect on bone marrow suppression induced by S-1 (Table 3).

**Table 2:** Univariate Cox proportional hazard model of bone marrow suppression induced by S-1

Variables	HR	95% CI	*p* value
Male gender	1.11	0.77–1.64	0.58
Age (years)	0.99	0.97–1.01	0.27
Height (cm)	1.01	0.99–1.03	0.57
Body weight (kg)	1.01	0.99–1.02	0.41
BMI (kg/m^2^)	1.02	0.97–1.07	0.50
BSA (m^2^)	1.50	0.53–4.19	0.44
Grade ≥ 2 leukopenia^a^	0.78	0.24–1.86	0.61
Grade ≥ 2 neutropenia^a^	0.66	0.20–1.61	0.39
Grade ≥ 2 lymphocytopenia^a^	3.79	1.31–8.78	< 0.05
Grade ≥ 2 anemia^a^	0.71	0.30–1.41	0.35
AST (IU/L)	1.00	0.99–1.01	0.91
ALT (IU/L)	1.00	0.99–1.01	0.76
Total bilirubin (mg/dL)	0.65	0.40–0.99	< 0.05
Serum albumin (g/dL)	0.78	0.50–1.24	0.29
eGFR^b^ (mL/min/1.73 m^2^)	1.00	0.99–1.01	0.98
Gastrointestinal cancer	1.09	0.71–1.64	0.68
Clinical cancer stage ≥III	0.99	0.64–1.60	0.97
RDI^c^ > 70%	1.54	0.99–2.48	< 0.05
RDI^c^ > 75%	1.52	1.05–2.21	< 0.05
RDI^c^ > 80%	1.39	0.97–1.99	0.07
Presence of combination chemotherapy	1.46	0.99–2.13	0.06
Prior history of cytotoxic chemotherapy	1.21	0.82–1.74	0.34

*HR* hazard ratio, *95% CI* 95% confidence interval, *BMI* body mass index, *BSA* body surface area, *AST* aspartate transaminase, *ALT* alanine aminotransferase, *eGFR* estimated glomerular filtration rate, *RDI* relative dose intensity

^a^Grade ≥ 2 leukopenia, neutropenia, lymphocytopenia, anemia, and thrombocytopenia at the initiation of S-1 treatment were assessed using Common Terminology Criteria for Adverse Events (CTCAE) version 4.0. No patient had Grade ≥ 3 thrombocytopenia.

^b^eGFR was calculated using a prediction equation.

^c^RDI is the ratio of the actual dose intensity administered and the planned dose intensity.

**Table 3:** Multivariate Cox proportional hazard model of bone marrow suppression induced by S-1

Variables	Model 1 (with RDI^b^ > 70%)			Model 2 (with RDI^b^ > 75%)			Model 3 (with RDI^b^ > 80%)		
	HR	95% CI	*p* value	HR	95% CI	*p* value	HR	95% CI	*p* value
Grade ≥ 2 lymphocytopenia^a^	3.93	1.35–9.11	< 0.05	5.17	1.72–12.6	< 0.01	4.75	1.60–11.4	< 0.01
RDI^b^ > 70%	1.42	0.88–2.38	0.16	–	–	–	–	–	–
RDI^b^ > 75%	–	–	–	1.71	1.10–2.72	< 0.05	–	–	–
RDI^b^ > 80%	–	–	–	–	–	–	1.65	1.07–2.54	< 0.05

*HR* Hazard ratio, *95% CI* 95% confidence interval, *RDI* relative dose intensity

^a^Grade ≥ 2 lymphocytopenia at the initiation of S-1 treatment was assessed using Common Terminology Criteria for Adverse Events (CTCAE) version 4.0.

^b^RDI is the ratio of the actual dose intensity to the planned dose intensity.

## Discussion

This study revealed that bone marrow suppression induced by S-1 was associated with not only grade ≥ 2 lymphocytopenia at baseline, but also RDI > 75% and RDI > 80%. RDI > 75 and > 80% is useful to identify patients at a high risk of developing grade ≥ 3 of bone marrow suppression by S-1. When we used grade ≥ 2 of bone marrow suppression as dependent variables, significant relationships between the outcomes and any clinical variables, including RDIs, could not be detected (data not shown). Therefore, our study results should be interpreted as predictors of an only severe bone marrow suppression by S-1.

Leucopenia and lymphocytopenia have been identified as predictors of bone marrow suppression for a number of cytotoxic chemotherapy regimens [14–16]. Moreover, lymphocytopenia is risk factors for bacteremia and the severity of clinical course in cancer patients [17]. Our results indicated that grade ≥ 2 lymphocytopenia at baseline is associated with bone marrow suppression induced by S-1, which are consistent with the results of previous studies on cytotoxic chemotherapy [15]. Furthermore, grade ≥ 1 leucopenia and neutropenia are associated with improved chemotherapeutic outcomes [18, 19]. However, lymphocytopenia is associated with poor chemotherapeutic outcomes [20]. Therefore, lymphocytopenia may be associated with the reduced efficacy and tolerability of S-1 treatment for unknown reasons.

Focusing on anti-tumor effect of fluoropyrimidine derivatives, RDI > 70 and > 89.5% have shown significantly better relapse-free survival in colon and gastric cancer compared with that of ≤70 and ≤ 89.5% [21, 22]. Our study demonstrated that patients with RDI > 70% did not have a high incidence of bone marrow suppression induced by S-1. Despite that patients categorized in the higher RDI had a higher rate of receiving combination chemotherapy in this study and that a combination chemotherapy could increase the risk of adverse drug events during chemotherapy in general, our stepwise Cox proportional hazard analyses failed to detect this factor as a significant independent variable of developing bone marrow suppression induced by S-1. Therefore, RDI of 70 to 75% may be a reasonable option for patients who cannot tolerate S-1irrespective of patient’s history and regimen of chemotherapy.

High RDI clinically correlates with better prognosis of various cancers [10, 14]. Focusing on S-1, RDI > 80% is associated with better prognosis [11]. Our study indicated that RDI > 75 and > 80% are associated with bone marrow suppression induced by S-1. This result was maintained when a median RDI (78.8%) was used as an independent variable instead of the RDIs of > 75 and > 80% (data are not shown). The incidence of bone marrow suppression induced by S-1 is reported to be higher in previous clinical trials than that observed in our study (> 20% vs 13.3%) [1–3], probably because median RDI is also higher in these previous studies than in our study (> 90% vs 78.8%). Kim et al. reported that a decreased RDI was related to poor disease-free survival in patients with stage II-IV gastric cancer and the hazard ratios for relapse and death in the S-1-completion group were significantly lower than those in the discontinuation group [23]. In addition, Kawazoe et al. reported that overdose of S-1 is associated with discontinuation of treatment [23]. Therefore, first dose of S-1 should be optimized to each patient to complete S-1 treatment. In our study, the cutoff value of RDI to prevent bone marrow suppression was 75%, thus it might be reasonable that we decide to give 75% RDI of S-1 as an initial dose for patients who do not require intensive S-1 treatment (e.g., adjuvant chemotherapy for stage I cancer).

There were several limitations in this study. First, this was a retrospective study and the sample size was limited. In addition, since data were recorded in electronic medical records, missing data was an inevitable limitation. Second, we did not analyze performance statuses and body temperatures, so the influence of performance status and the incidence of febrile neutropenia could not be assessed. Third, although subsets of lymphocytes are associated with cytotoxic chemotherapy-induced neutropenia [24], there were no data on lymphocyte subsets in our study population. Fourth, the other definitions of RDI used in other clinical studies [25, 26] (i.e., 85, 90, and 95%) could not be assessed because the number of patients with RDI > 85% was limited. Fifth, we studied a limited number of patients with renal failure. Thus, it was difficult to evaluate the relationship between renal function and bone marrow suppression induced by S-1. Finally, our study did not include any data on genetic polymorphisms that influence the efficacy and tolerability of S-1.

## Conclusions

In summary, grade ≥ 2 lymphocytopenia and high RDI have a significant impact on bone marrow suppression induced by S-1. Further study is needed to evaluate the influence of RDI considering the risk-benefit profile of S-1 treatment.

## Abbreviations

95% CI: 95% confidence interval
ALC: Absolute lymphocyte count
ALT: Alanine aminotransferase
ANC: Absolute neutrophil count
AST: Aspartate transaminase
BMI: Body mass index
BSA: Body surface area
CTCAE: Common terminology criteria for adverse events
eGFR: Estimated glomerular filtration rate
Hb: Hemoglobin
HR: Hazard ratio
IQR: Interquartile range
Plt: Platelet count
RDI: Relative dose intensity
S-1: A combination of tegafur, gimeracil, and oteracil
SD: Standard deviation
WBC: White blood cell count

## References

[1] Koizumi W, Narahara H, Hara T, Takagane A, Akiya T, Takagi M (2008). S-1 plus cisplatin versus S-1 alone for first-line treatment of advanced gastric cancer (SPIRITS trial): a phase III trial. Lancet Oncol..

[2] Yamada Y, Takahari D, Matsumoto H, Baba H, Nakamura M, Yoshida K (2013). Leucovorin, fluorouracil, and oxaliplatin plus bevacizumab versus S-1 and oxaliplatin plus bevacizumab in patients with metastatic colorectal cancer (SOFT): an open-label, non-inferiority, randomised phase 3 trial. Lancet Oncol.

[3] Okamoto I, Yoshioka H, Morita S, Ando M, Takeda K, Seto T (2010). Phase III trial comparing oral S-1 plus carboplatin with paclitaxel plus carboplatin in chemotherapy-naive patients with advanced non-small-cell lung cancer: results of a West Japan oncology group study. J Clin Oncol.

[4] Shirasaka T, Nakano K, Takechi T, Satake H, Uchida J, Fujioka A (1996). Antitumor activity of 1 M tegafur-0.4 M 5-chloro-2,4-dihydroxypyridine-1 M potassium oxonate (S-1) against human colon carcinoma orthotopically implanted into nude rats. Cancer Res.

[5] Shirasaka T, Shimamato Y, Ohshimo H, Yamaguchi M, Kato T, Yonekura K (1996). Development of a novel form of an oral 5-fluorouracil derivative (S-1) directed to the potentiation of the tumor selective cytotoxicity of 5-fluorouracil by two biochemical modulators. Anti-Cancer Drugs.

[6] Shirasaka T, Shimamoto Y, Fukushima M (1993). Inhibition by oxonic acid of gastrointestinal toxicity of 5-fluorouracil without loss of its antitumor activity in rats. Cancer Res.

[7] Fujita K, Yamamoto W, Endo S, Endo H, Nagashima F, Ichikawa W (2008). CYP2A6 and the plasma level of 5-chloro-2, 4-dihydroxypyridine are determinants of the pharmacokinetic variability of tegafur and 5-fluorouracil, respectively, in Japanese patients with cancer given S-1. Cancer Sci.

[8] Ikeda M, Furukawa H, Imamura H, Shimizu J, Ishida H, Masutani S (2002). Pharmacokinetic study of S-1, a novel oral fluorouracil antitumor agent in animal model and in patients with impaired renal function. Cancer Chemother Pharmacol.

[9] Bender BC, Schindler E, Friberg LE (2015). Population pharmacokinetic–pharmacodynamic modelling in oncology: a tool for predicting clinical response. Br J Clin Pharmacol.

[10] Yabusaki N, Fujii T, Yamada S, Murotani K, Sugimoto H, Kanda M (2016). The significance of relative dose intensity in adjuvant chemotherapy of pancreatic ductal adenocarcinoma-including the analysis of clinicopathological factors influencing relative dose intensity. Medicine (Baltimore).

[11] Kitagawa M, Shimura T, Yamada T, Ebi M, Hirata Y, Mizoshita T (2012). The relationship between antitumor effects and relative dose intensity of S-1 plus cisplatin treatment for metastatic gastric cancer. Anticancer Res.

[12] Matsuo S, Imai E, Horio M, Yasuda Y, Tomita K, Nitta K (2009). Revised equations for estimated GFR from serum creatinine in Japan. Am J Kidney Dis.

[13] Common Terminology Criteria for Adverse Events (CTCAE) v4.0. https://ctep.cancer.gov/protocolDevelopment/electronic_applications/ctc.htm#ctc_40. Accessed 31 July 2018.

[14] Lyman GH, Kuderer NM, Crawford J, Wolff DA, Culakova E, Poniewierski MS (2011). Predicting individual risk of neutropenic complications in patients receiving cancer chemotherapy. Cancer.

[15] Ray-Coquard I, Borg C, Bachelot T, Sebban C, Philip I, Clapisson G (2003). Baseline and early lymphopenia predict for the risk of febrile neutropenia after chemotherapy. Br J Cancer.

[16] Ray-Coquard I, Le Cesne A, Rubio MT, Mermet J, Maugard C, Ravaud A (1999). Risk model for severe anemia requiring red blood cell transfusion after cytotoxic conventional chemotherapy regimens. The Elypse 1 study group. J Clin Oncol.

[17] Zahorec R (2001). Ratio of neutrophil to lymphocyte counts--rapid and simple parameter of systemic inflammation and stress in critically ill. Bratisl Lek Listy.

[18] Pallis AG, Agelaki S, Kakolyris S, Kotsakis A, Kalykaki A, Vardakis N (2008). Chemotherapy-induced neutropenia as a prognostic factor in patients with advanced non-small cell lung cancer treated with front-line docetaxel-gemcitabine chemotherapy. Lung Cancer.

[19] Yamanaka T, Matsumoto S, Teramukai S, Ishiwata R, Nagai Y, Fukushima M (2007). Predictive value of chemotherapy-induced neutropenia for the efficacy of oral fluoropyrimidine S-1 in advanced gastric carcinoma. Br J Cancer.

[20] Lissoni P, Brivio F, Fumagalli L, Messina G, Ghezzi V, Frontini L (2004). Efficacy of cancer chemotherapy in relation to the pretreatment number of lymphocytes in patients with metastatic solid tumors. Int J Biol Markers.

[21] Aspinall SL, Good CB, Zhao X, Cunningham FE, Heron BB, Geraci M (2015). Adjuvant chemotherapy for stage III colon cancer: relative dose intensity and survival among veterans. BMC Cancer.

[22] Kim SJ, Kim YJ, Kim JH, Park DJ, Kim HH, Lee JS (2013). Safety, compliance, and predictive parameters for dosage modification in adjuvant S-1 chemotherapy for gastric cancer. Cancer Sci.

[23] Kawazoe H, Shimasaki M, Ueno M, Sumikawa S, Takatori S, Namba H (2015). Risk factors for discontinuation of s-1 adjuvant chemotherapy for gastric cancer. J Cancer.

[24] Borg C, Ray-Coquard I, Philip I, Clapisson G, Bendriss-Vermare N, Menetrier-Caux C (2004). CD4 lymphopenia as a risk factor for febrile neutropenia and early death after cytotoxic chemotherapy in adult patients with cancer. Cancer.

[25] Yokoyama M, Kusano Y, Takahashi A, Inoue N, Ueda K, Nishimura N (2017). Incidence and risk factors of febrile neutropenia in patients with non-Hodgkin B-cell lymphoma receiving R-CHOP in a single center in Japan. Support Care Cancer.

[26] Zhang L, Yu Q, Wu XC, Hsieh MC, Loch M, Chen VW (2018). Impact of chemotherapy relative dose intensity on cause-specific and overall survival for stage I-III breast cancer: ER+/PR+, HER2- vs. triple-negative. Breast Cancer Res Treat.

